# Amyloid precursor-like protein 2 expression in macrophages: differentiation and M1/M2 macrophage dynamics

**DOI:** 10.3389/fonc.2025.1570955

**Published:** 2025-04-08

**Authors:** Gabrielle L. Brumfield, Shelby M. Knoche, Kenadie R. Doty, Alaina C. Larson, Brittany J. Poelaert, Don W. Coulter, Joyce C. Solheim

**Affiliations:** ^1^ Eppley Institute, University of Nebraska Medical Center, Omaha, NE, United States; ^2^ Fred & Pamela Buffett Cancer Center, University of Nebraska Medical Center, Omaha, NE, United States; ^3^ Department of Pediatrics, University of Nebraska Medical Center, Omaha, NE, United States; ^4^ Children's Nebraska, Omaha, NE, United States

**Keywords:** amyloid precursor-like protein 2, cancer, differentiate, inflammation, macrophage, M1, M2, neuroblastoma

## Abstract

Amyloid precursor-like protein 2 (APLP2) has been previously associated with pro-tumor phenotypes in cancer cells, and in this current study we investigated the expression and functions of this protein in macrophages. Our findings showed that APLP2 expression was increased in monocyte-like U937 cells after cytokine-induced differentiation to macrophage-like cells. Evaluation of human mRNA data revealed that APLP2 is more highly expressed in human M2/anti-inflammatory (pro-tumor) macrophages than in M1 macrophages (which have a pro-inflammatory, anti-tumor phenotype). Consistent with the mRNA data, by immunoblotting we identified increased APLP2 protein expression in mouse M2/anti-inflammatory macrophages. Intratumoral infiltration of M2/anti-inflammatory macrophages has been reported in several cancers, including neuroblastoma (NB). We observed that treatment of macrophages with NB-conditioned media induced M2/anti-inflammatory and mixed phenotypes. Through comparison of macrophages from wild-type and APLP2-knockout mice, we correlated alterations in inflammation-associated markers with the presence of APLP2. This suggests that APLP2 influences macrophage polarization dynamics between M0/unpolarized and pro- and anti-inflammatory states, and populations altered by APLP2 KO resemble the macrophage profiles altered with NB-conditioned media treatment. In total, our work implicates APLP2 as a mediator of macrophage status, namely in the M0/unpolarized macrophage and the M1/pro-inflammatory and M2/anti-inflammatory axis.

## 1 Introduction

Amyloid precursor-like protein 2 (APLP2) is a type I transmembrane protein and member of the amyloid precursor protein (APP) family, which also includes APP and amyloid precursor-like protein 1 (APLP1) (1–4). Proteolytic processing of APP family members generates cleavage fragments, such as the APP-derived amyloid-β fragment implicated in Alzheimer’s disease. APLP1 and APLP2 demonstrate homology to APP in sequence and domain architecture, though only APP has the specific amyloid-β sequence associated with Alzheimer’s disease. APLP1 expression is restricted to cells of the nervous system, but APP and APLP2 are broadly expressed (2, 3, 5–9).

Analyses in various model systems have shown that APLP2 mediates adhesion, signaling, migration, and mitosis (10–16). Studies from our laboratory on cancer cell lines demonstrated that APLP2 associates with the major histocompatibility complex (MHC) class I molecule, which presents tumor antigens and thereby activates T cell killing of malignant cells (17–20). In cancer cells, APLP2 binds MHC class I at the cell surface and facilitates its internalization and routing to the lysosomes (17–19, 21, 22).

Abnormal expression of APLP2 has been reported in several types of cancer. For example, Ewing’s sarcoma, glioblastoma, pancreatic cancer, and gastrointestinal neuroendocrine cell cancers all overexpress APLP2 (21–28). Studies in glioblastoma showed a positive correlative relationship between high APLP2 expression and reduced overall survival (23). APLP2 expression was noted to be low in benign thyroid follicular lesions, but higher in malignant lesions (29). Our laboratory has previously shown that Ewing’s sarcoma cell lines’ expression of APLP2 increases their resistance to radiation and lysis by immune cells (22). Similarly, in colon cancer cells, APLP2 was associated with suppression of apoptosis and enhancement of cellular proliferation (27, 30). Additional work by our research group showed that APLP2 is highly expressed in pancreatic cancer cells, and its presence in these cells increases their migration and growth, facilitates pancreatic tumor metastasis, and shortens the survival of pancreatic tumor-bearing mice (28). In contrast, APLP2 expression was reduced in clear cell renal cell carcinoma, and low APLP2 levels corresponded to worse patient outcomes, suggesting that APLP2 may have a dynamic role that varies among cancer types (31).

Reports characterizing APLP2’s function in macrophages are limited. THP-1 monocyte-like cells that have been differentiated to macrophages produce proteoglycans that include APLP2 as a major component, suggesting a role for APLP2 in the extracellular matrix (32). Additional studies in monocytes identified both APP and APLP2 as binders of tissue inhibitor of metalloproteinases 1 (TIMP-1), though association of only APP, not APLP2, with TIMP-1 induces inflammatory cytokine release (33). In another study, evaluation of macrophage-related gene expression revealed that APLP2 is one component of a nine-gene prognostic signature in hepatocellular carcinoma patients, where an increased risk score correlated with higher tumor stage, tumor grade, and mortality (34). APLP2 knockout (KO) was found to downregulate the production of inducible nitric oxide synthase (iNOS) and IL-1β, thus interfering with macrophage killing of *Mycobacterium tuberculosis* (*M. tuberculosis*) (35). Furthermore, mouse *M. tuberculosis*-infected macrophages have lower expression of the *Aplp2* gene than uninfected macrophages. Together, these results suggest that M. tuberculosis can limit the presence of APLP2 in macrophages as a strategy to evade the host’s immune response (35).

Macrophages play a complex role in cancer, where they may aid in elimination of the tumor, generally through M1/pro-inflammatory mechanisms, or perpetuate tumor growth with M2/anti-inflammatory activity. Compared to M1/pro-inflammatory macrophages, M2/anti-inflammatory macrophages show enhanced migration, decreased antigen presentation, and increased secretion of immune-dampening factors (36–38). Suppressing immune responses, stimulating angiogenesis, augmenting drug resistance, and facilitating metastasis are M2 macrophage pro-tumor effects observed in neuroblastoma (NB) and other types of cancers (39–42). Within NB, leukocyte infiltrate is often dominated by T cells in the early disease process, and patients that retain this phenotype have favorable prognosis (37, 43). However, as NB tumors progress, an immunosuppressive infiltrate of anti-inflammatory macrophages overrules, and survival rates decline (37). These immunosuppressive macrophages may have originated as tumor-reactive and later experienced internal and environmental signals that prompted their phenotypic switch; however, the mechanisms governing the transition and resultant profiles of these cells are not well understood. Although there are reports of APLP2 having some roles in macrophages (as described above), there is an absence of information regarding how APLP2 may contribute to the development of the unfavorable M2/anti-inflammatory phenotype (such as is observed in NB). Our study seeks to discern the role of APLP2 in the immunosuppressive macrophage, with the goal to further understand macrophage physiology and the NB immune environment. This knowledge will serve as a foundation for developing future therapeutic strategies for cancers, such as NB, that exhibit increased intratumoral infiltration of M2/anti-inflammatory macrophages.

## 2 Materials and methods

### 2.1 Cell lines

The following cell lines were utilized in these studies: the U937 monocyte-like histiocytic lymphoma cell line, the L-929 mouse fibroblast cell line, and the mouse-derived 9464D NB cell line. U937 was used as a cell model for monocyte-to-macrophage development, as it is known to transition to a macrophage-like phenotype following exposure to phorbol 12-myristate 13-acetate (PMA) (44). For monocyte maturation, U937 cells were cultured in 100 ng/mL PMA (MilliporeSigma, Burlington, MA, USA) for 24 hours and monitored for adherence and morphological qualities characteristic of macrophages. The culture medium for both U937 and L-929 cells was Life Technologies RPMI 1640 (Thermo Fisher Scientific, Waltham, MA, USA) and 9464D cells were cultured in Dulbecco’s Modified Eagle Medium (Thermo Fisher Scientific). Culture media for all cell lines was supplemented with the following to generate complete media: 10% fetal bovine serum (R&D Systems, Minneapolis, MN, USA) that had been heat inactivated at 56°C for 30 minutes, 2 mM L-glutamine, 1 mM sodium pyruvate, 10 mM HEPES, 1x non-essential amino acids, 100 mg/mL streptomycin, 100 units/mL penicillin (all purchased from Thermo Fisher Scientific). All cell lines were maintained in a humidified 37°C, 5% CO_2_ cell incubator.

### 2.2 Antibodies

The antibodies used for Western blotting recognized GAPDH (Cell Signaling Technology, Danvers, MA, USA), HSC70 (Enzo Life Sciences, Farmingdale, NY, USA), full-length APLP2 (R&D Systems), C-terminal cleavage fragment of APLP2 (custom-made antiserum; Thermo Fisher Scientific), iNOS (Novus Biologicals, Centennial, CO, USA), and arginase 1 (Arg1) (Cell Signaling Technology, Danvers, MA, USA). For flow cytometry, F4/80 (Miltenyi Biotec, Bergisch Gladbach, Rhineland, Germany) and CD11b (Miltenyi Biotec) were used for identification of murine macrophages, and iNOS (Thermo Fisher Scientific), CD68 (BioLegend, San Diego, CA, USA) and Arg1 (Thermo Fisher Scientific), CD206 (BioLegend) antibodies were used as markers for M1/pro-inflammatory macrophages and M2/anti-inflammatory macrophages, respectively.

### 2.3 mRNA data analysis

Human monocyte-derived macrophage mRNA data (series GSE5099) in the National Center for Biotechnology Information Gene Expression Omnibus (NCBI GEO), previously cited by Martinez et al. and Solinas et al., were analyzed for APLP2 expression in monocytes, as well as M0/unpolarized, M1/pro-inflammatory, and M2/anti-inflammatory macrophage populations (45, 46).

### 2.4 Generation of L-929 conditioned media

L-929 cell line culture supernatant contains macrophage colony stimulating factor (M-CSF), which acts as media additive to promote the generation of macrophages from mouse bone marrow cells. For collection of L-929 supernatant, the cells were plated in 600 mL tissue culture flasks (VWR, Radnor, PA, USA) containing 50 mL complete medium. Cells were plated at a density expected to reach 100% confluency within 1-2 days and incubated for a total of 14 days prior to media removal, filter sterilization, and storage of the generated supernatant at -80°C until use.

### 2.5 Mouse genotyping

APLP2-KO mice on the C57BL/6 background were produced by crossing C57BL/6 mice with cre recombinase under the EIIa adenovirus promoter [B6.FVB-Tg(EIIa-cre)C5379Lmgd/J] (The Jackson Laboratory, Bar Harbor, ME, USA) with previously described floxed *Aplp2* mice (28). Polymerase chain reaction was used to confirm cre (forward primer: 5’-CCTGGAAAATGCTTCTGTCCG-3’, reverse primer: 5’-CAGGGTGTTATAAGCAATCCC-3’) and *Aplp2* (forward primer: 5’-ATTATTAGACTTGGCAGGCATGCTG-3’, wild-type reverse primer: 5’-ACATTTCCTGGCTACAATCCTGTGC-3’, floxed reverse primer: 5’-GATGCCTTCTTGGGAGCTCTGC-3’) status for KO of *Aplp2*. Mouse housing, handling, and experimental procedures were conducted in accordance with University of Nebraska Medical Center (UNMC) Institutional Animal Care and Use Committee (IACUC) protocols 17-141-12-EP (breeding) and 18-011-02-EP (experimental). Mice were euthanized by CO_2_ inhalation using a flow rate of 30-70% of the chamber volume per minute, and the method used to ensure death was cervical dislocation.

### 2.6 Bone marrow-derived macrophage isolation and polarization

Post euthanasia, mouse fibulas and tibias were excised, and one epiphysis of each bone was removed using a pair of sharp scissors. With the exposed marrow cavity pointed downward, bones were placed in 0.5-mL microcentrifuge tubes that had previously been punctured on the bottom by a 20G needle. After closing the lid, the 0.5-mL microcentrifuge tube was nested inside a 1.5-mL microcentrifuge tube containing 150 μL phosphate-buffered saline (PBS, Thermo Fisher Scientific), and the nested tubes were spun in a 4°C cooled Eppendorf 5417R (Eppendorf, Framingham, MA, USA) centrifuge at 10,000 revolutions per minute (RPM) for 1 minute. Isolated bone marrow contents were washed with 1 mL cold PBS and transferred to a 15 mL conical tube, after which an additional 10 mL PBS was added prior to centrifugation in a 4°C cooled Eppendorf 5810R centrifuge at 1,200 RPM for 5 minutes. Within a sterile cell culture hood, the PBS wash was aspirated and red blood cells were lysed by addition and gentle trituration of 900 μL sterile water (Thermo Fisher Scientific), followed quickly by addition and gentle mixing of 100 μL 10x PBS (Thermo Fisher Scientific). The cells were then filtered through a 70-μm cell strainer (Thermo Fisher Scientific) into a 50-mL conical tube and centrifuged in a 4°C cooled Eppendorf 5810R centrifuge at 1,200 RPM for 5 minutes. After removal of the supernatant, isolates were resuspended in RPMI 1640 (Thermo Fisher Scientific) complete medium supplemented with 10% L-929-conditioned media. Isolates were cultured in L-929-supernatant-containing media for 7 days to induce monocyte differentiation to macrophages. On day 7, the cells were further cultured in 1 of 3 conditions: 10 ng/mL lipopolysaccharide (LPS) + 20 ng/mL recombinant mouse interferon-γ (IFN-γ) (BioLegend) to generate M1 (pro-inflammatory) macrophages, 10 ng/mL recombinant mouse IL-4 (BioLegend) to cause differentiation to M2 (anti-inflammatory) macrophages, or in L-929-conditioned medium for M0 (undifferentiated) macrophages.

### 2.7 Flow cytometry

For use in flow cytometry assays, cells were detached from culture flasks by treatment with TrypLE™ Express Enzyme (Thermo Fisher Scientific). The cells were suspended in complete medium and pelleted by centrifugation in an Eppendorf 5810R centrifuge at 1,500 RPM for 5 minutes at 4°C. After centrifugation, cells were suspended at a concentration of 2 x 10^6^ cells/100 μL in PBS, and 100 μL aliquots of the cell suspension were placed into wells of a 96-well plate. The plate was centrifuged at 1,500 RPM for 5 minutes at 4°C, and after the supernatants were removed, cells were suspended in TruStain FcX (Biolegend) Fc receptor blocking solution for reduction of off-target antibody binding. After 15 minutes of incubation, cells are centrifuged, supernatant is removed, and cells are resuspended in PBS with primary surface antibodies for macrophage selection (CD11b, F4/80), LIVE/DEAD Fixable Blue Dead Cell Stain (ThermoFisher Scientific), and extracellular markers of macrophage phenotype (CD68, CD206) prior to incubation at 4°C for 30 minutes. Cells were then subjected to a cycle of centrifugation at 1,500 RPM for 5 minutes, followed by washing in PBS. Centrifugation and washing were repeated 3 times. Following, cells were fixed in 2% paraformaldehyde. For intracellular staining, cells were then permeabilized via incubation in permeabilization buffer (0.5% Tween 20 [Thermo Fisher Scientific] in PBS) for 15 minutes at room temperature. Staining for intracellular antigens (iNOS and Arg1) was performed with the same procedure as surface antigens, though intracellular antibodies were diluted in permeabilization buffer. Flow cytometric analysis of stained cells was performed at the University of Nebraska Medical Center Flow Cytometry Research Facility on a BD LSR II Flow Cytometer (BD Biosciences, Franklin Lakes, NJ, USA). Resulting flow cytometry data was analyzed with FlowJo™ (v.10.10.0).

### 2.8 Immunoblotting

Cells harvested for immunoblotting experiments were lysed with RIPA buffer (Thermo Fisher Scientific) containing 2 mM dithiothreitol (MilliporeSigma), 0.1 mM phenylmethylsulfonyl fluoride (MilliporeSigma), 1 mM Na_3_VO_4_, and 1 mg/mL Halt Cocktail (Thermo Fisher Scientific). The cell lysates were frozen at -80°C overnight, thawed, and centrifuged at 13,000 RPM for 30 minutes at 4°C in an Eppendorf 5417R centrifuge. Aliquots of the supernatants were mixed with 5x loading dye (10% w/v sodium dodecyl sulfate [Bio-Rad Laboratories, Hercules, CA, USA], 30% v/v glycerol, 250 mM Tris-HCl pH 6.8, 0.2% w/v bromophenol blue and 5% v/v β-mercaptoethanol [all purchased from MilliporeSigma]). Before loading onto Invitrogen Novex Tri-glycine polyacrylamide pre-cast gels (Thermo Fisher Scientific), the samples were boiled for 5 minutes at 95°C. Samples were electrophoresed for 2 hours at 100 volts and the proteins were then transferred at 30 volts for 1 hour and 50 minutes onto an Immobilon-P Millipore polyvinylidene difluoride membrane (MilliporeSigma, Burlington, MA, USA). A room-temperature solution of 5% w/v nonfat dry milk was used for membrane blocking to avoid binding of antibodies to non-specific sites, and the primary antibody was added to the milk solution before overnight incubation of the membrane in the solution at 4°C. The next day, the membrane was washed in 0.1% Tween-20 (Thermo Fisher Scientific) in pH 7.4 Tris-buffered saline, using 3 room-temperature washes of 5 minutes in length. The secondary antibody was added and incubated with the membrane at room temperature for 1 hour, and then the membrane was washed 3 times as described above. The membrane was incubated at room temperature for 3 minutes in Pierce ECL Western Blotting Substrate (Thermo Fisher Scientific) and visualization was done with the Bio-Rad ChemiDoc Imaging System B and Image Lab software (v.6.1).

### 2.9 Statistical analysis

GraphPad Prism version 10.2.3 was utilized for conducting statistical testing and creating graphical visuals. Significance was determined by p value < 0.05, and p values are represented in graphics as *p<0.05, **p<0.01, ***p<0.001, and ****p<0.0001. The statistical test used for each experimental set is indicated in the corresponding figure legend. When warranted, multiple comparisons were accounted for by the use of Tukey’s multiple comparisons test.

## 3 Results

### 3.1 APLP2 expression increases in matured monocyte-derived macrophages

**Figure 1:**
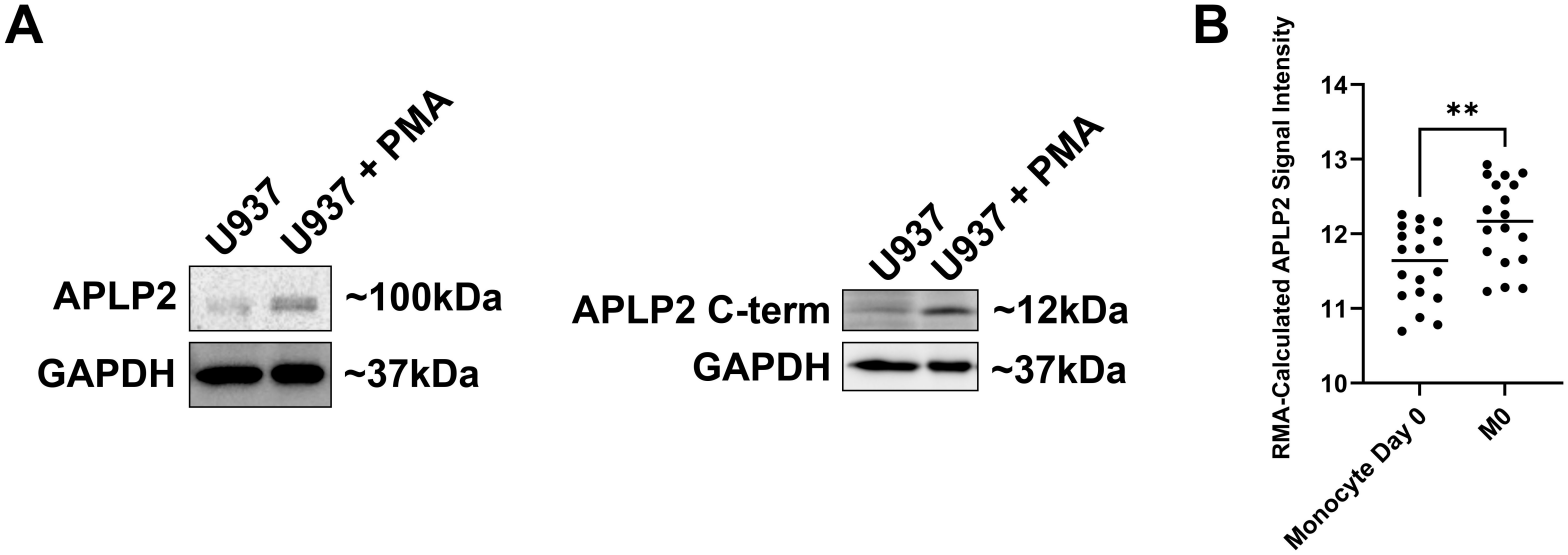
APLP2 and APLP2 C-terminal fragment are upregulated in mature macrophages. **(A)** U937 monocyte-like histiocytic lymphoma cell lysates were harvested post 24-hour incubation in 100 ng/mL PMA for differentiation to a macrophage-like phenotype. A non-PMA-treated U937 population was utilized as a control. Western blots were probed for full-length APLP2 (n=3, *left panel*) and APLP2 C-terminal fragment (n=2, *right panel*) with GAPDH included as a loading control. **(B)** GEO microarray data series GSE5099 was queried for APLP2 mRNA expression in human monocytes (monocyte day 0) or macrophages matured by 7-day treatment with 100 ng/mL M-CSF (M0). Results represent mean robust multi-array analysis (RMA)-calculated signal intensity of six APLP2 probes detected across each sample. Statistical significance was determined by unpaired t-test (n=3). **p<0.01.

To evaluate APLP2 expression during the processes of macrophage development, we first cultured U937 monocyte-like cells in medium with PMA (100 ng/mL for 24 hours). Exposure to PMA caused the U937 cells to undergo differentiation to macrophage-like cells (detectable by observing cell adherence, as well as slight enlargements in size and spreading of pseudopodia). APLP2 protein expression increased following macrophage differentiation in PMA-treated U937 cells compared to undifferentiated U937 cells (**Figure 1A**, *left panel*). APLP2 can undergo intracellular cleavage by β-secretase that produces a C-terminal fragment of ~12 kDa, and this APLP2 C-terminal fragment was also found to be elevated in PMA-treated U937 cells (**Figure 1A**, *right panel*). Evaluation of APLP2 mRNA in untreated human monocytes and M-CSF-matured macrophages demonstrated a similar trend with increased expression of APLP2 mRNA in mature macrophages (**Figure 1B**).

### 3.2 APLP2 is decreased in M1/pro-inflammatory macrophages and increased in M2/anti-inflammatory macrophages

**Figure 2:**
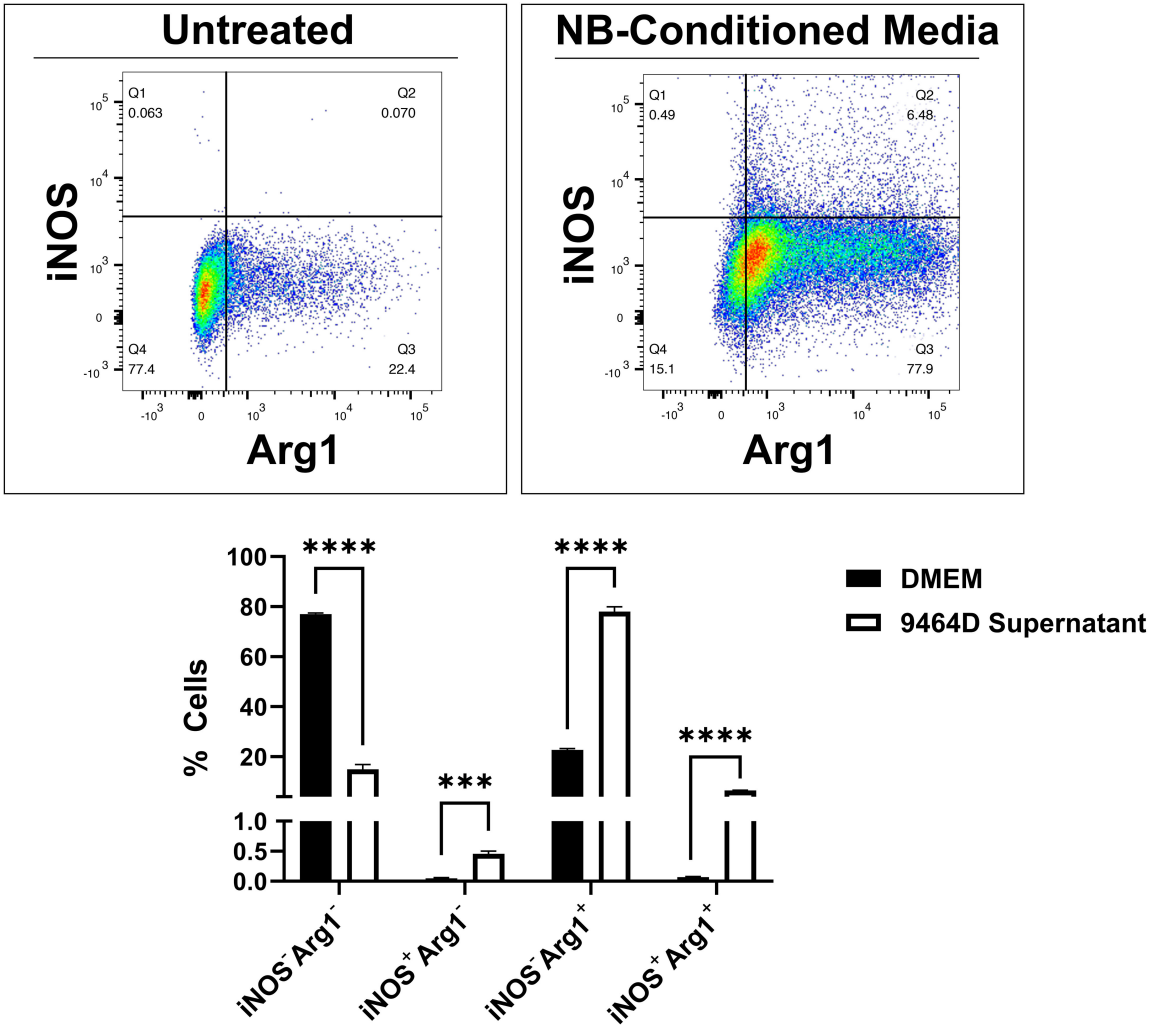
Murine macrophages treated with NB tumor cell culture supernatant are polarized toward the M2/anti-inflammatory phenotype. Primary macrophages were collected from C57BL/6 mice by treatment of tibia and fibula bone marrow with M-CSF-containing L-929-conditioned media for 7 days. Concurrently, the NB cell line 9464D was cultured for 48 hours to reach 70% confluency, after which the culture supernatant was collected. Macrophages were treated with DMEM (*top left*) or 9464D-conditioned media (*top right*) for 24 hours prior to harvest and flow cytometry analysis with the pro-inflammatory marker iNOS and the anti-inflammatory marker Arg1. Quantified results (*bottom*) represent mean number of positive cells ± SEM. Statistical significance was determined by unpaired t-test (n=3). ***p<0.001, ****p<0.0001.

Macrophages with an M2/anti-inflammatory phenotype tend to have pro-tumor effects; this has been observed in NB as well as other types of cancers (36, 37). Tumors may perpetuate the detrimental influences of M2 macrophages, as tumor cells can direct macrophages to adopt an M2 phenotype, thus creating a cascading, pro-tumor cycle. We observed this effect for NB cells, as primary mouse macrophages exposed to NB tumor cell culture supernatant predominantly displayed polarization to the M2/anti-inflammatory (iNOS^-^Arg1^+^) phenotype (**Figure 2** and **Supplementary Figure 1**). A smaller increase was observed in macrophages displaying mixed M1/M2 markers (iNOS^+^Arg1^+^), and no significant increase in the anti-tumoral M1/pro-inflammatory population (iNOS^+^Arg1^-^).

**Figure 3:**
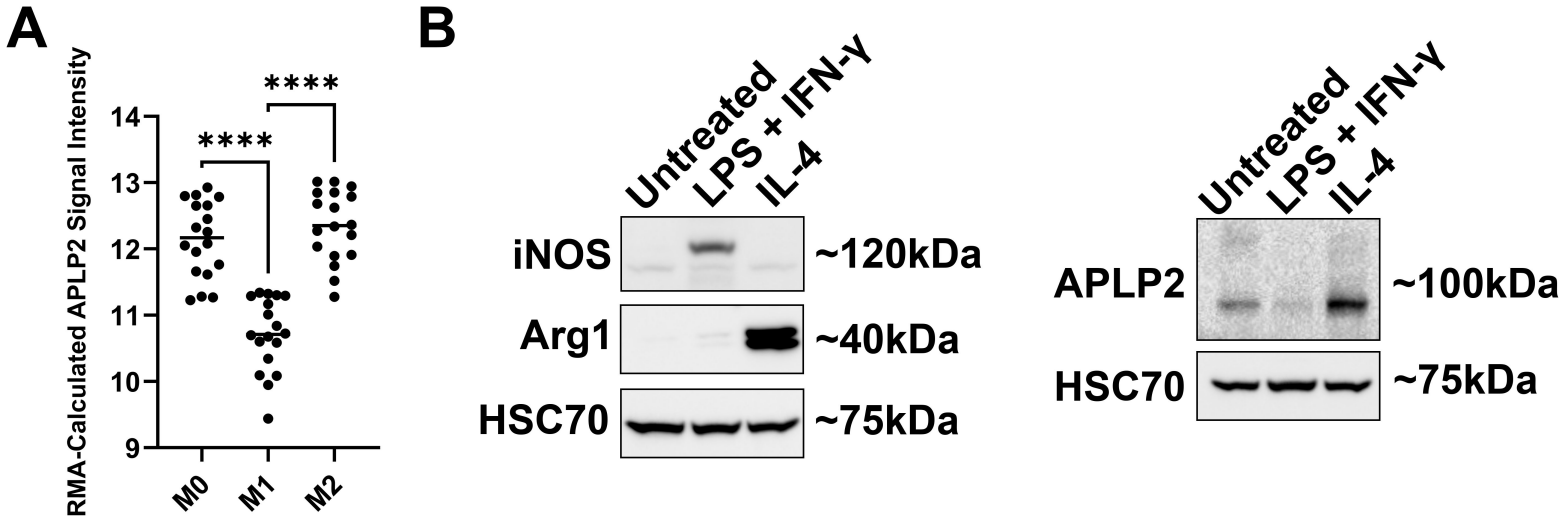
M2 macrophages have higher expression of APLP2 than M1 macrophages. **(A)** The graph displays data from the NCBI GEO data set series GSE5099. Human monocytes were treated with 100 ng/mL M-CSF for 7 days to promote differentiation into macrophages (M0). Mature macrophages were then treated with either LPS + IFN-γ or with IL-4 to generate M1 or M2 macrophages, respectively. Results represent mean ± SEM. Statistical significance was determined by one-way ANOVA with Tukey’s multiple comparisons test (n=3). ****p<0.0001 **(B)** Femur and tibia bone marrow samples from C57BL/6 mice were treated with L-929-conditioned medium for 7 days prior to treatment with the indicated cytokines for 24 hours. M1/pro-inflammatory macrophages were generated with 10 ng/mL LPS + 20 ng/mL IFN-γ, and M2/anti-inflammatory macrophages were induced with 20 ng/mL IL-4. Whole cell lysates were probed by western blot for iNOS and Arg1 for confirmation of macrophage phenotypes (*left panel*) and for APLP2 expression (*right panel*) (n=3).

For evaluation of APLP2 expression in macrophages with an M0/unpolarized, M1/pro-inflammatory, or M2/anti-inflammatory phenotype, we first assessed human NCBI GEO mRNA data. As shown by mRNA microarray, APLP2 expression is significantly higher in human M2/anti-inflammatory macrophages compared to M1/pro-inflammatory macrophages (**Figure 3A**). In addition, M1/pro-inflammatory macrophages were found to have significantly lower expression of APLP2 mRNA relative to M0/unpolarized macrophages (**Figure 3A**).

Following, for analysis of whether APLP2 expression is also elevated in murine M2/anti-inflammatory macrophages, we first cultured femur and tibia bone marrow from C57BL/6 mice with L-929-conditioned media for 7 days to produce mature macrophages. We next induced macrophage transition to either the M1/pro-inflammatory phenotype (via 24-hour treatment with 10 ng/mL LPS + 20 ng/mL IFN-γ) or to the M2/anti-inflammatory subset (via 24-hour treatment with 20 ng/mL IL-4). Immunoblotting of macrophage lysates for iNOS (pro-inflammatory marker), Arg1 (anti-inflammatory marker), and HSC70 (loading control) validated their expected differentiation in response to the cytokine treatments (**Figure 3B**, *left panel*). To evaluate APLP2 expression in the differentiated macrophages, we immunoblotted for APLP2 (or for HSC70, as a control) and observed that M2/anti-inflammatory macrophages expressed more APLP2 than the M1/pro-inflammatory macrophages (**Figure 3B**, *right panel*). Furthermore, lower levels of APLP2 were observed in M1/pro-inflammatory macrophages relative to M0/unpolarized macrophages (**Figure 3B**, *right panel*). Thus, APLP2 expression levels correlate with the pro- or anti-inflammatory status of macrophages.

### 3.3 APLP2 facilitates M0 and mixed macrophage phenotypes

**Figure 4:**
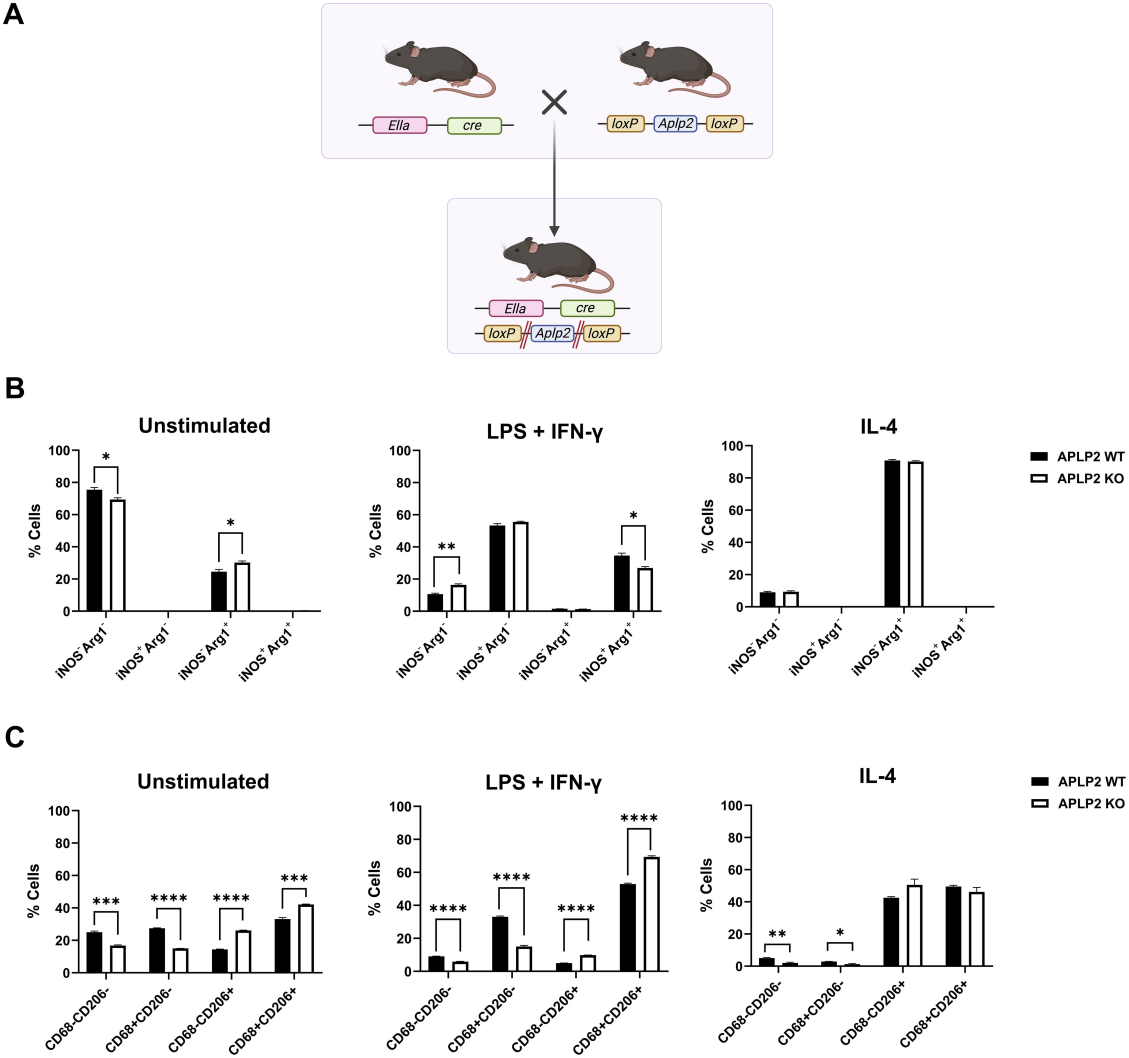
APLP2 KO shifts macrophage subpopulations at baseline and post pro-inflammatory stimulation. **(A)** APLP2-KO mice were generated by breeding C57BL/6 mice expressing cre recombinase housed under the EIIa adenovirus promoter with mice containing floxed *Aplp2*. Genotypes of offspring were verified by PCR for *Aplp2* and EIIa-cre. **(B, C)** Bone marrow samples isolated from femurs and tibias of APLP2-WT C57BL/6 mice and APLP2-KO mice were treated with media supplemented with L-929-conditioned media as an M-CSF source. The generated macrophages were left unstimulated as a control (*left*) or stimulated with 10 ng/mL LPS + 20 ng/mL IFN-γ (pro-inflammatory stimuli, 6 hours, *middle*) or with 10 ng/mL IL-4 (anti-inflammatory stimuli, 24 hours, *right*) for determination of **(B)** iNOS/Arg1 and **(C)** CD68/CD206 expression. Results represent mean ± SEM. Statistical significance was determined by unpaired t-test (n=3). *p<0.05, **p<0.01, ***p<0.001, ****p<0.0001.

The correlation between APLP2 expression and macrophage inflammatory states raised the question of whether APLP2’s presence or absence can influence the polarization of macrophages to the M0 (iNOS^-^Arg1^-^, CD68^-^CD206^-^), M1 (iNOS^+^Arg1^-^, CD68^+^CD206^-^), or M2 (iNOS^-^Arg1^+^, CD68^-^CD206^+^) phenotypes. To address this question, we utilized APLP2-KO mice (**Figure 4A**) in addition to syngeneic C57BL/6 mice (referred to here as APLP2-WT mice).

Macrophages derived from the bone marrow of APLP2-WT and APLP2-KO mice were polarized with 20 ng/mL IL-4 or with 10 ng/mL LPS + 20 ng/mL IFN-γ. These cells were identified as macrophages by flow cytometry detection of CD11b and F4/80 and were further stratified into macrophage phenotypes by iNOS/Arg1 and CD68/CD206 staining (**Figures 4B, C** and **Supplementary Figure 2**). In unstimulated APLP2-KO macrophages, there was a statistically significant decrease in the percentage of M0 cells (iNOS^-^Arg1^-^, CD68^-^CD206^-^) as well as a significant increase in the percentage of M2 cells (iNOS^-^Arg1^+^, CD68^-^CD206^+^) (**Figures 4B, C**, *left panels*). These data suggest that the presence of APLP2 facilitates the M0 phenotype in preference to the M2 phenotype. For the LPS + IFN-γ-treated macrophages from APLP2-KO mice, M0 and double positive populations were affected for both sets of phenotypic markers (iNOS^+^Arg1^+^, CD68^+^CD206^+^) (**Figures 4B, C**, *middle panels*). These double-positive macrophages represent a potential intermediate state, and the observation that the loss of APLP2 affects this intermediate population again suggests that APLP2 may influence the ability of macrophages to transition into a stable phenotype. For the IL-4-stimulated macrophages, a high proportion from both the APLP2-WT and APLP2-KO mice were M2 macrophages (iNOS^-^Arg1^+^, CD68^-^CD206^+^), and there were no significant differences in the M2 percentages between APLP2-WT and APLP2-KO (**Figures 4B, C**, *right panels*). The lack of effect from APLP2 KO on M2 differentiation suggests that although APLP2 is highly expressed in Arg1^+^ M2 macrophages (**Figure 3B**), it is not required for the expression of Arg1 and CD206 in the M2 cells.

## 4 Discussion

Because changes in macrophage inflammatory status drive immune dysfunction in cancer, autoimmunity, and infectious pathology, there is clinical need to identify molecular factors contributing to macrophage development and activity. In this study, we sought to determine the expression and role of APLP2 in maturing macrophages and pro- vs anti-inflammatory subpopulations. Our findings indicate higher APLP2 expression is associated with mature macrophages and anti-inflammatory subpopulations (**Figures 1**, **3**). Additionally, APLP2 is implicated in the maintenance of unpolarized macrophage populations and the generation of mixed phenotype macrophages (**Figure 4**).

Significant shedding of APLP2 as a potential component of the extracellular matrix has been associated with differentiation of monocytes to mature macrophages, though the degree of full-length and cleaved APLP2 retained by cells during the transition was unclear (32). *In vitro*, we observed increased cell-associated full-length APLP2 in PMA-matured U937 cells compared to untreated controls (**Figure 1A**, *left panel*). Additionally, there was an increased presence of the cleaved C-terminal tail (**Figure 1A**, *right panel*). Analysis of mRNA data from human monocytes and M-CSF-matured macrophages reflected this same trend with increased APLP2 mRNA detected in macrophages (**Figure 1B**). Given APLP2’s association with migration of multiple cell types, retained APLP2 may facilitate the altered adhesion and migration mechanisms utilized by monocytes transversing the epithelial barrier and, after maturing to macrophages, navigating the tissue extracellular matrix. Our findings provide additional context to APLP2 trafficking and processing in the maturing monocyte-derived macrophage.

Predominance of M2 over M1 macrophages is reported in many cancers, and in NB, there is an observed shift toward M2 intratumoral infiltrates with disease progression (37). It remains unclear whether these cells are new infiltrates polarized to an M2 state or M1 cells that have shifted phenotype. In evaluation of these NB-associated macrophages, treatment with NB-conditioned media predominantly induced an M2/anti-inflammatory phenotype (**Figure 2**). The majority of cells shifted to an iNOS^-^Arg1^+^ population, with a smaller though significant accumulation of double-positive iNOS^+^Arg1^+^ cells, and a minority of cells single positive for iNOS (**Figure 2**). Though the predominant population is iNOS^-^Arg1^+^ cells, these phenotypic shifts do not fully replicate population alterations observed in macrophages treated with purely pro- or anti-inflammatory stimuli (**Figures 2**, **4B**), demonstrating the multiplex effects of cancer on the surrounding immune environment and the need to further understand these largely M2 cancer-associated macrophages.

Further evaluation of M2/anti-inflammatory macrophages revealed increased APLP2 expression compared to M1/pro-inflammatory macrophages, observed in both protein of primary murine macrophages and mRNA of human macrophages (**Figure 3**). In analysis of APLP2’s role in expression of the inflammatory mediators iNOS and Arg1 in addition to the M1/M2 markers CD68 and CD206 through APLP2 KO, at baseline APLP2-KO macrophages had a higher proportion of M2 cells compared to WT control (**Figures 4B, C**, *left panels*). These data suggest that the presence of APLP2 facilitates the M0 phenotype in preference to the M2 phenotype, which was unexpected due to higher APLP2 expression found in M2 macrophages. Similarly, when macrophages were challenged with inflammatory stimuli for 6 hours (previously determined to produce peak iNOS expression), there was no significant shift in the single-positive M1 population between WT and APLP2-KO groups (**Figure 4B**, *middle panel*). Earlier studies associated decreased iNOS mRNA in bulk analysis of peritoneal macrophages isolated from APLP2 mutant mice exposed to *M. tuberculosis* (35). In our analysis of individual cells by flow cytometry, there is in fact an overall decrease in percentage of iNOS^+^ cells (iNOS^+^Arg1^-^ or iNOS^+^Arg1^+^), though no significant change in the single-positive iNOS^+^Arg1^-^ M1 population (**Figure 4B**, *middle panel*). Instead, the predominant loss of iNOS^+^ cells was through a decrease in the mixed iNOS^+^Arg1^+^ cells. These decreased double-positive iNOS^+^Arg1^+^ cells then appeared to accumulate as an unpolarized iNOS^-^Arg1^-^ phenotype. This phenomenon may reveal a more complex role of APLP2 in regulation of inflammatory macrophage phenotype beyond the enhancement of inflammatory signaling that has been attributed to APP family members (33, 35). Double-positive cells may display a mixed phenotype or, alternatively, may be transitioning between anti- and pro-inflammatory states. KO of APLP2 leading to a decrease in this mixed population shows potential for APLP2 to play a role in the macrophage phenotypic switch or mixed populations, such as those that were observed in the macrophages treated with NB-conditioned media. These data, in combination with literature associating APP family members with enhanced inflammation, suggest that APLP2 may have a role in initial upregulation of inflammatory mediators, leading to a hastened transition of macrophages to their anti-inflammatory counterpart or depolarization to an M0 state. Promoting mixed or M0/undifferentiated macrophages in preference to the anti-tumor M1/pro-inflammatory macrophages implicates a role for APLP2 in impeding an effective anti-tumor immune response. Reduction of pro-tumoral macrophages in the tumor microenvironment has shown to improve anti-tumoral immune responses (47, 48). Additionally, infiltration of pro-inflammatory macrophages is associated with improved outcomes in multiple cancer types, thus highlighting the importance of macrophages subtypes to the anti-tumor immune response (49–51). Therefore, modulation of macrophage phenotypes through alterations to APLP2 could act as a therapeutic target for altering macrophage phenotype without complete removal of this myeloid subset. Given that APLP2 expression is altered in many cancer cells and APLP2 expression is correlated with poor outcomes, APLP2-targeted therapy might have potential to benefit cancer therapy through immune-mediated, as well as non-immune-mediated, mechanisms. However, particularly since the observed differences shown in **Figure 4** are rather mild, it also remains possible that APLP2’s biological impact in macrophages is relatively minor, and so further functional studies will be needed to clarify the extent of APLP2’s physiological importance in these immune cells.

Notably, despite our association of higher APLP2 expression with M2/anti-inflammatory macrophages, there were no significant shifts in iNOS/Arg1 and CD68/CD206 expression in IL-4-stimulated APLP2-KO cells. While APLP2 may not play a role in iNOS/Arg1 and CD68/206 expression in this population, APLP2 may play an indirect role in inflammatory mediation. Both APP and APLP2 were found to bind TIMP-1 in monocytes, though only binding of APP triggered inflammatory cytokine release. In this case, APLP2 may act as a competitive binding partner to TIMP-1, thus shrinking the pool of inflammatory molecules available for association with APP and decreasing pro-inflammatory potential of M2 macrophages. Additional studies have linked APLP2 to increased cell migration and decreased MHC surface expression, both characteristic of anti-inflammatory macrophages (8, 10–12, 17, 19–21, 25). The C-terminal tail of APLP2 may also traffic to the nucleus and act in conjunction with the transcriptional regulator Fe65 and transcription factors, regulating downstream processes such as apoptosis, migration, and DNA repair (52–55). APLP2 may play a role in transcriptional regulation of macrophage phenotype, or even as a regulator of macrophage persistence. Therefore, APLP2 presence or absence could affect the survival of macrophage subsets predisposed for polarization to specific subtypes rather than directly affecting the process of polarization. The APP protein may also act through similar Fe65-binding mechanisms to affect transcription, and in the setting of APLP2 KO, has the potential to compensate for lack of APLP2-mediated transcriptional regulation (52, 53). Though beyond the scope of this study, investigation of APLP2’s role in macrophage function outside the production of inflammatory mediators is needed for elucidating the complex functionality of APLP2 modulation of macrophages.

In summary, our results implicate APLP2 as a potentiator of M0/unpolarized macrophage status and development of macrophages with mixed inflammatory markers. This study further clarifies the expression of APLP2 in macrophage subpopulations, in addition to expanding the knowledge of macrophage subtypes associated with the NB tumor environment and our understanding of APLP2’s role in macrophage physiology.

## Data Availability

The original contributions presented in the study are included in the article/**Supplementary Material**. Further inquiries can be directed to the corresponding author.
