# Supplementary figures and images for: Amyloid precursor-like protein 2 expression in macrophages: differentiation and M1/M2 macrophage dynamics
Source: Front Oncol. 2025 Apr 8;15:1570955. doi: 10.3389/fonc.2025.1570955 (PMC12011594; doi:10.3389/fonc.2025.1570955)

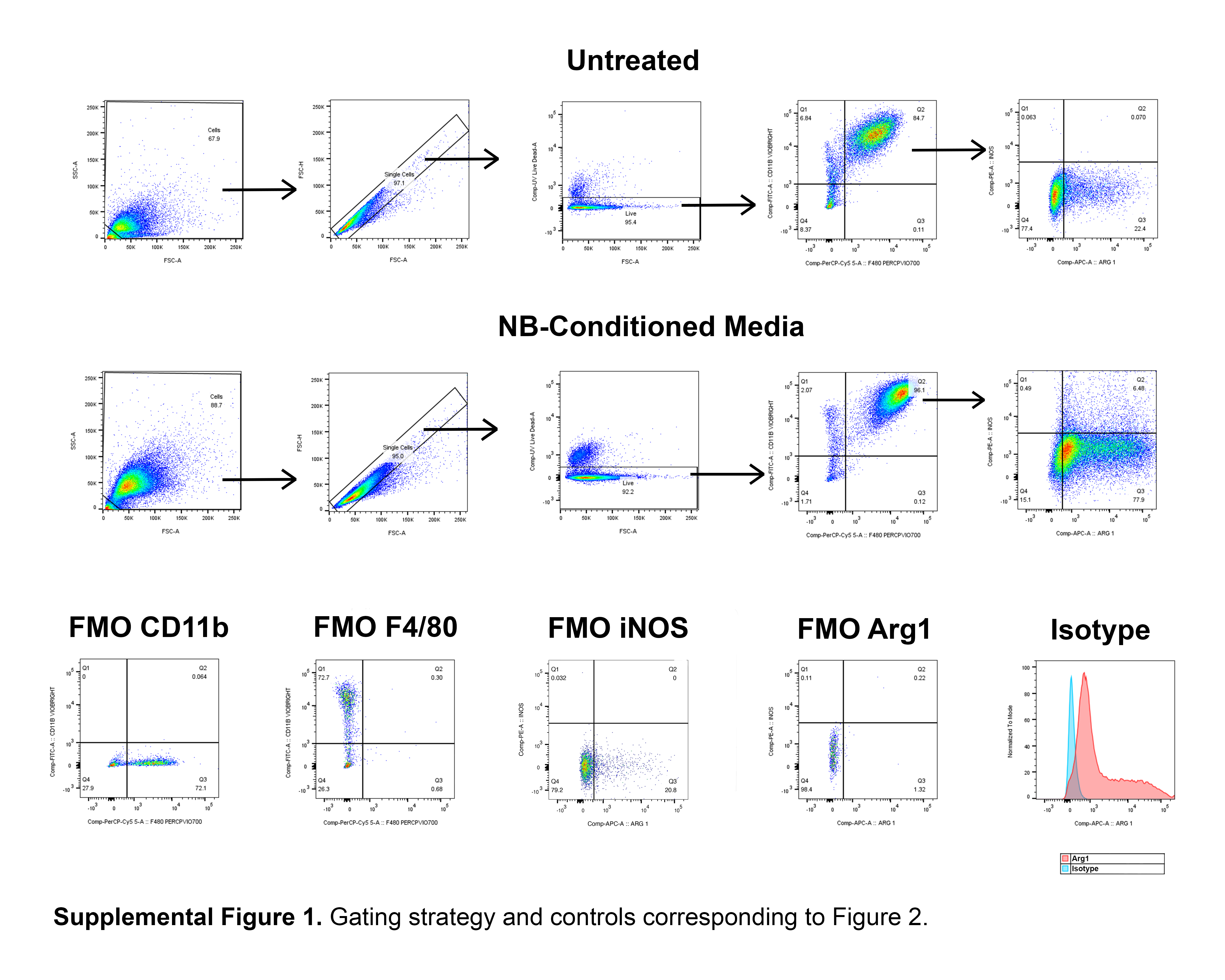

Supplement: Supplementary file 1 [file Image1.tif]

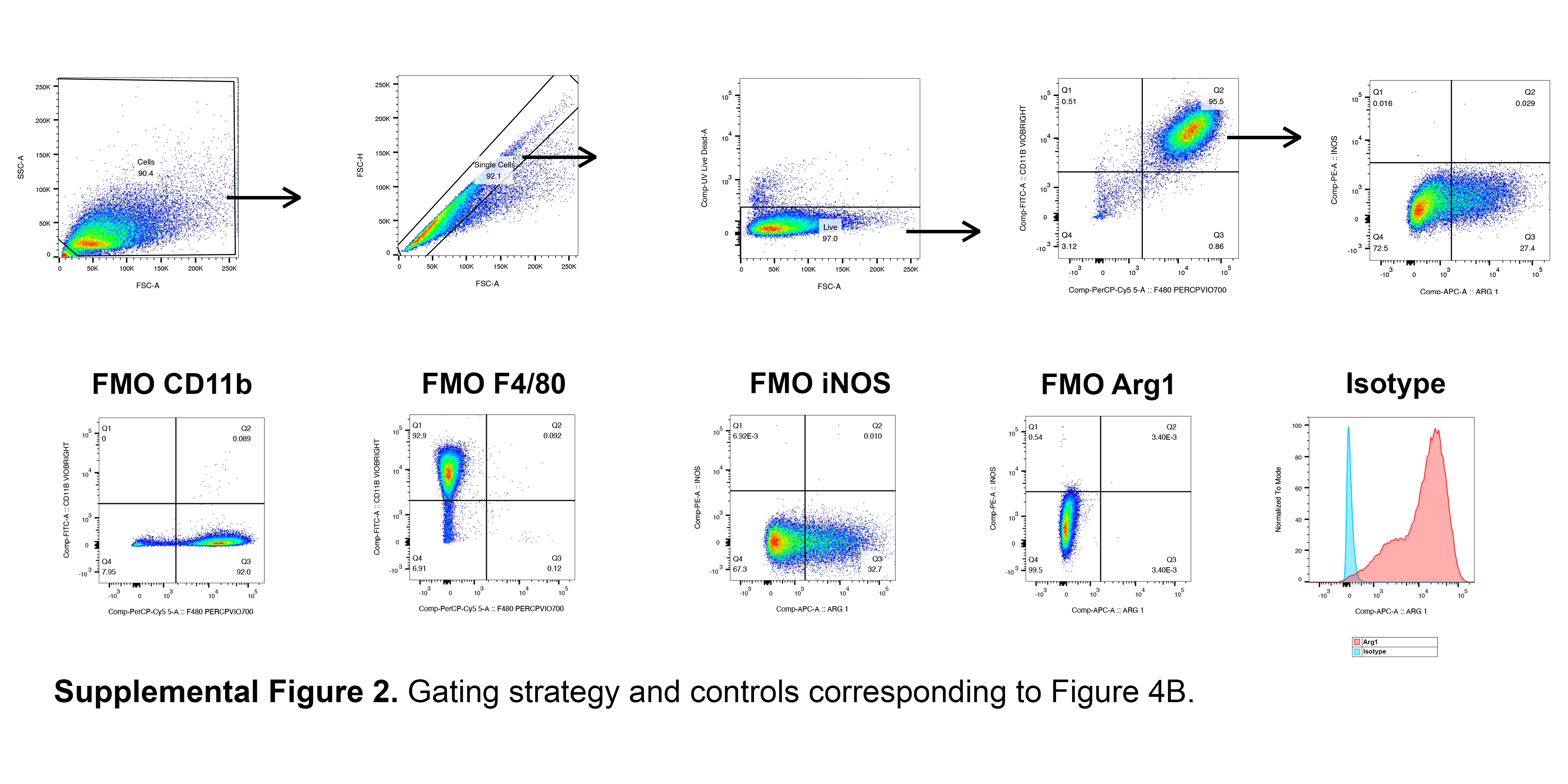

Supplement: Supplementary file 2 [file Image2.tif]
